# A Novel Human Microbe-Disease Association Prediction Method Based on the Bidirectional Weighted Network

**DOI:** 10.3389/fmicb.2019.00676

**Published:** 2019-04-09

**Authors:** Hao Li, Yuqi Wang, Jingwu Jiang, Haochen Zhao, Xiang Feng, Bihai Zhao, Lei Wang

**Affiliations:** ^1^Key Laboratory of Hunan Province for Internet of Things and Information Security, Xiangtan University, Xiangtan, China; ^2^Clinical Lab, Yongcheng People's Hospital, Shangqiu, China; ^3^College of Computer Engineering & Applied Mathematics, Changsha University, Changsha, China

**Keywords:** microbe, disease, association prediction, bidirectional weighted network, bidirectional recommendations

## Abstract

The survival of human beings is inseparable from microbes. More and more studies have proved that microbes can affect human physiological processes in various aspects and are closely related to some human diseases. In this paper, based on known microbe-disease associations, a bidirectional weighted network was constructed by integrating the schemes of normalized Gaussian interactions and bidirectional recommendations firstly. And then, based on the newly constructed bidirectional network, a computational model called BWNMHMDA was developed to predict potential relationships between microbes and diseases. Finally, in order to evaluate the superiority of the new prediction model BWNMHMDA, the framework of LOOCV and 5-fold cross validation were implemented, and simulation results indicated that BWNMHMDA could achieve reliable AUCs of 0.9127 and 0.8967 ± 0.0027 in these two different frameworks respectively, which is outperformed some state-of-the-art methods. Moreover, case studies of asthma, colorectal carcinoma, and chronic obstructive pulmonary disease were implemented to further estimate the performance of BWNMHMDA. Experimental results showed that there are 10, 9, and 8 out of the top 10 predicted microbes having been confirmed by related literature in these three kinds of case studies separately, which also demonstrated that our new model BWNMHMDA could achieve satisfying prediction performance.

## 1. Introduction

Microorganisms are small in shape, simple in structure, and closely related to human beings. The development of modern bioinformatics and sequencing technologies has led to the study of microorganisms living in the ocean, soil, human body, and other places by the scientific community (Gilbert and Dupont, 2011). Among them, eukaryotes, archea, bacteria, and viruses are human-related microorganisms, collectively known as human microbiota (Turnbaugh et al., 2007; Methé et al., 2012). Microorganisms exist in large quantities in humans, nearly 10 times that of human cells (Sender et al., 2016). According to recent researches, there are nearly 1,014 bacterial cells in the human body with more than 10,000 kinds of microorganisms, which provide different degrees of metabolic activity (Bhavsar et al., 2007; Turnbaugh et al., 2007; Shah et al., 2016). Parasitic in the human body, these microbes do not harm the host, but are interdependent with human beings and are called “forgotten organs” (Quigley, 2013). With the continuous advancement of high-throughput sequencing technology and analytical systems, people have gradually realized the importance of microorganisms in the investigation. According to the survey, microbes participate in a series of human life activities, such as harvesting and storing energy, regulating the immune system, protecting the human body from foreign microorganisms and pathogens, participating in the digestion and absorption of carbohydrates and promoting metabolism (Guarner and Malagelada, 2003; Gill et al., 2006). Therefore, once the microbes become “unhealthy” in the human body, the human body will receive their effects leading to physiological disorders and even illness.

Humans and commensal microbiota have formed a close symbiotic relationship in the process of continuous evolution. The microbiota will be affected by the host and living environment. It has been reported that diet affects the structure and activity of human intestinal microbes (Duncan et al., 2006; Ley et al., 2006; Walker et al., 2010; David et al., 2013) For example, a short-term high-fat, low-fiber diet can cause changes in microbial structure, while long-term diets are associated with alternative intestinal status (Wu et al., 2011). Besides, smoking (Mason et al., 2014), age, and genes are also factors influencing the composition of the microbiota (Gill et al., 2006). Therefore, once the human body and the microbiota cannot coexist harmoniously, it may cause various problems in the human body. Based on the 16S ribosomal RNA (rRNA) gene sequence and classification spectrum (Thompson et al., 2014; Jesmok et al., 2016), researchers have found that a large number of human diseases are closely related to human microorganisms, including cancer (Moore and Moore, 1995), diabetes (Wen et al., 2008; Brown et al., 2011; Qin et al., 2012), Obesity (Ley et al., 2005; Zhang et al., 2009), kidney stones (Hoppe et al., 2011), and other thorny diseases. For example, Huang (2013) pointed out that microbes can affect allergic sensitization and asthma development in susceptible individuals, and early intervention in promoting “healthy” human microbiome constitution may have the potential and benefits of preventing asthma. Hence, some researchers are proposing to promote the induction of sensitized immune response through the research and development of probiotic-based therapies (Rauch and Lynch, 2012).

Disease-related microbes are obtaining more and more attention from humans, and researchers have carried out some large-scale sequencing projects, including the Human Microbiome Project (HMP) (Turnbaugh et al., 2007) and the Earth Microbiome Project (EMP) (Gilbert et al., 2010). Moreover, some databases (Matsumoto et al., 2005; Faith et al., 2007; Chen et al., 2010; Mikaelyan et al., 2015) for categorizing and managing disease-related microbial information have also been developed. For instance, Ma et al. collected and compiled 483 pairs of human microbe-disease associations by collecting published literature and established the Human Microbe-Disease Association Database (HMDAD) (Ma et al., 2016). These accurate data provide the possibility to predict human microbes and diseases. Nowadays, most microbial community identification methods are independent culture methods and quantitative methods. Their shortcomings are obvious and often take a lot of time and efforts. Previously, many researchers have studied the potential correlation predictions of diseases and other biological categories (such as miRNA Chen and Yan, 2014; You et al., 2017; Chen et al., 2018b,c and lncRNA Chen and Yan, 2013; Chen et al., 2016b, 2018a; Yu et al., 2018; Xuan et al., 2019), and simultaneously, Drug-target interaction prediction (Chen et al., 2012) and the study of synergistic drug combinations prediction (Chen et al., 2016a) has also achieved satisfying successes. And among existing state-of-the-art methods, the computational model of KATZ measure for human microbe-disease association prediction (KATZHMDA) (Chen et al., 2017) proposed by Chen et al. is one of their prominent representatives, which not only achieved excellent prediction performance but also initialized the research field of the microbe-disease prediction. Later, Huang Z.A. et al. (2017) proposed a Path-Based computational model of Human Microbe-Disease Association prediction (PBHMDA), which adopts a special depth-first search algorithm to traverse all possible paths between microbes and diseases in heterogeneous networks to obtain the prediction score of each microbe-disease pair. Wang et al. (2017) proposed a semi-supervised learning-based computational model of Laplacian Regularized Least Squares for Human Microbe-Disease Association prediction (LRLSHMDA), which utilizes Laplace's regular least squares classification combined with topological information of the known microbe-disease association network to train an optimal classifier. Huang Y.A. et al. (2017) developed a method based on Neighbor and Graph-based combined recommendation model for Human Microbe-Disease Association prediction (NGRHMDA) by combining two recommendation models as a neighbor-based collaborative filtering model and a topology-based model. Peng et al. (2018) developed a model of Adaptive Boosting for Human Microbe-Disease Association prediction (ABHMDA), which reveals the associations between disease and microbe by using a strong classifier to calculate the probability of disease-microbe pair association. In addition, Shen et al. (2018) proposed Bi-Random Walk based on Multiple Path (BiRWMP) to predict microbe-disease associations. Shi et al. (2018) propose BMCMDA based on Binary Matrix Completion to predict potential microbe-disease associations.

In this paper, inspired by the performance of KATZHMDA, we proposed a new microbe-disease association prediction model called BWNMHMDA. A novel two-way network was constructed firstly based on the known microbe-disease associations downloaded from the HMDAD database, and then, the Gaussian interaction profile kernel similarity were adopted to assign weights to every node and edge in a newly constructed two-way network. Hence, a bidirectional weighted network was further obtained by implementing two newly developed bidirectional recommendation measures. Finally, based on the newly constructed bidirectional weighted network, a computational model was constructed to infer potential microbe-disease associations. In order to estimate the prediction performances of BWNMHMDA, the framework of leave-one-out cross validation (LOOCV) and 5-fold cross validation(5-Fold CV) were implemented, and simulation results indicated that BWNMHMDA could achieve reliable AUCs of 0.9127 in LOOCV and 0.8967 ± 0.0027 in 5-Fold CV, respectively, which is much better than that of state-of-the-art methods. And moreover, in case studies of asthma, colorectal carcinoma, and chronic obstructive pulmonary disease, the simulation results also demonstrated the effective predictability of BWNMHMDA.

## 2. Material

Since known microbe-disease associations were considered in our prediction model BWNMHMDA, we firstly downloaded known microbe-disease associations from the Human Microbe-Disease Association database (HMDAD) (Ma et al., 2016), and as a result, after getting rid of the redundant associations, a total of 450 different microbe-disease associations including 39 human diseases and 292 microbes were collected from 61 public publications. Hence, a 39 × 292 dimensional adjacency matrix *A* is obtained finally, which will be utilized as the data source of our prediction model BWNMHMDA. And additionally, in the adjacency matrix *A*, the value of *A*[*i*][*j*] is set to 1 if there is a known association between the *i*th disease and the *j*th microbe, otherwise, *A*[*i*][*j*] is set to 0.

## 3. Methods

**Figure 1:**
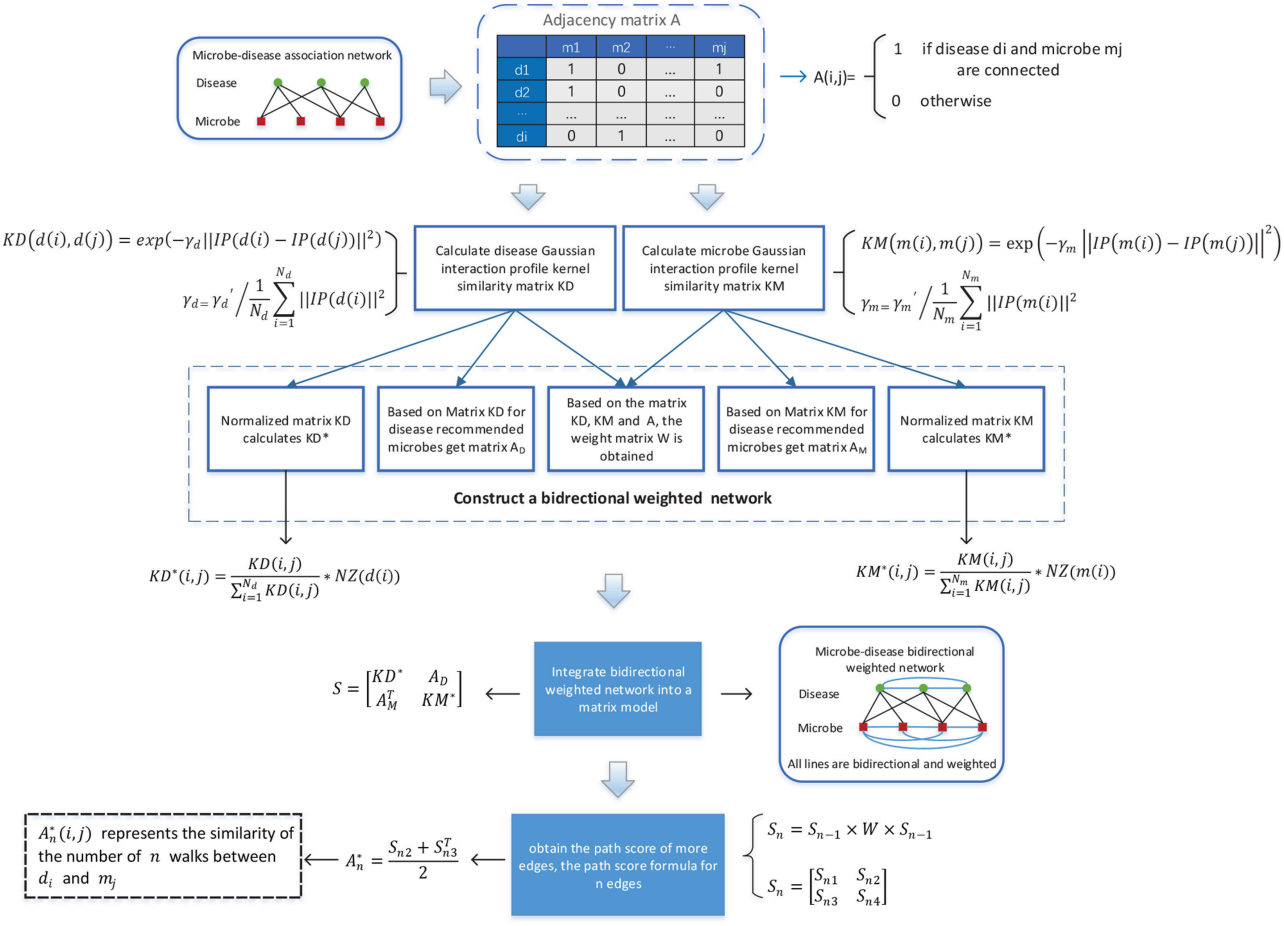
Flowchart of BWNMHMDA.

As illustrated in the following Figure 1, in BWNMHMDA, three kinds of association networks such as the known microbe-disease association network, the microbe similarity network and the diseases similarity network will be constructed firstly. And then, through integrating these three kinds of association networks, an integrated microbe-disease heterogeneous association network will be obtained. Moreover, through adopting the Gaussian interaction profile kernel similarity to assign weights to every node and edge in the integrated microbe-disease heterogeneous association network, a bidirectional weighted microbe-disease association network can be further obtained. Hence, based on the newly constructed bidirectional weighted association network, a novel computation model can be developed to infer potential microbe-disease associations.

### 3.1. Microbes Similarity Based on Gaussian Interaction Profile Kernel Similarity

It is obviously reasonable that for any two microbes if there are more common human diseases proved to be related to them, may tend to share more functional similarities potentially. Hence, in the known microbe-disease association network, we will first adopt the Gaussian interaction profile kernel similarity to construct a microbe similarity network according to the following formula (1):

(1) $$\begin{array}{r} KM\left(m\left(i\right),m\left(j\right)\right)=\exp\left(-\gamma_{m}||IP\left(m\left(i\right)\right)-IP\left(m\left(j\right)\right)|{|}^{2}\right) \end{array}$$

Where *m*(*i*) and *m*(*j*) represent the *i*th and *j*th microbes respectively in the adjacency matrix *A*, *IP*[*m*(*i*)] and *IP*[*m*(*j*)] denote *i*th and *j*th column, respectively, in the adjacency matrix *A*, and ||*X*|| represents the norm of the vector *X*. Moreover, the parameter γ_*m*_ can be obtained as follows:

(2) $$\gamma_{m}=\gamma_{m}{}^{\prime }/\frac{1}{N_{m}}\sum_{i=1}^{N_{m}}{\left\|IP(m(i))\right\|}^{2}$$

Here, $\gamma_{m}\prime$ is a parameter utilized to control the Gaussian kernel bandwidth, and according to the related studies (van Laarhoven et al., 2011), $\gamma_{m}\prime$ will be set to 1 in BWNMHMDA. In addition, the parameter *N*_*m*_ indicates the total number of microbes collected from the HMDAD database, and it is obvious that there is *N*_*m*_=292.

Thereafter, according to the above formula (1), it is easy to see that a microbe similarity matrix *KM* can be calculated, specifically, and for simplicity, we will replace *KM*[*m*(*i*), *m*(*j*)] with *KM*(*i, j*) in the following sections.

### 3.2. Diseases Similarity Based on Gaussian Interaction Profile Kernel Similarity

In a similar way, through adopting the Gaussian interaction profile kernel similarity, we can further construct a disease similarity network according to the following formula (3):

(3) $$\begin{array}{r} KD\left(d\left(i\right),d\left(j\right)\right)=\exp\left(-\gamma_{d}||IP\left(d\left(i\right)\right)-IP\left(d\left(j\right)\right)|{|}^{2}\right) \end{array}$$

Here, the parameter γ_*d*_ can be obtained as follows:

(4) $$\gamma_{d}=\gamma_{d}{}^{\prime }/\frac{1}{N_{d}}\sum_{i\;=\;1}^{N_{d}}{\left\|IP(d(i))\right\|}^{2}$$

Here, $\gamma_{d}\prime$ is a parameter utilized to control the Gaussian kernel bandwidth, and according to the related studies (van Laarhoven et al., 2011), $\gamma_{d}\prime$ will be also set to 1. In addition, the parameter *N*_*d*_ indicates the total number of diseases collected from the HMDAD database, and it is obvious that there is *N*_*d*_=39.

Thereafter, according to the above formula (3), it is easy to see that a disease similarity matrix *KD* can be calculated, specifically, and for simplicity, we will replace *KD*[*d*(*i*), *d*(*j*)] with *KD*(*i, j*) in the following sections.

### 3.3. Data Pre-processing

Based on the newly constructed microbe similarity network and disease similarity network, after integrating the known microbe-disease associations with these two similarity networks, it is obvious that we can construct an integrated heterogeneous microbe-disease association network consisting of two kinds of nodes such as microbe and disease, and three kinds of edges such as the edges between microbes, the edges between microbes and diseases, and the edges between diseases. And furthermore, based on the integrated heterogeneous microbe-disease association network, we can obtain a (39+292) × (39+292) dimensional matrix *P* as follows:

(5) $$\begin{array}{r} p=\left[\begin{array}{cc} KD & A\\ A^{T} & KM \end{array}\right] \end{array}$$

Moreover, in the integrated heterogeneous microbe-disease association network, if a microbe (or disease) node has more edges connecting with disease (or microbe) nodes, then it is obvious that the microbe (or disease) node will have less significance to those disease (or microbe) nodes connecting with it, which means that the microbe (or disease) node shall be assigned smaller weights than those microbe (or disease) nodes with fewer edges. Hence, based on above formula (5), we can further obtain a (39+292) × (39+292) dimensional diagonal matrix *W* to represent the weight value of each node in the heterogeneous network as follows:

(6) $$\begin{array}{r} W=diag\left(1/\left(P\times P^{T}\right)\right) \end{array}$$

In addition, while calculating the similarity between two nodes in the heterogeneous network, there may be cases where the scores of the path consisting of three edges are larger than the scores of the path consisting of two edges. Hence, in order to avoid such kind of situation, we will normalize the weights of edges in the heterogeneous network by adopting the following formula (7) and formula (8) separately.

(7) $$KM^{*}(i,j)=\frac{KM(i,j)}{\sum_{i=1}^{N_{m}}KM(i,j)}\times NZ(m(i))$$

Where *NZ*[*m*(*i*)] denotes the number of elements with non-zero values in the *i*th row of the matrix *KM*. And based on above formula (7), it is noteworthy that the symmetric matrix *KM* will be changed to an asymmetric matrix KM^*^ after the normalization. Moreover, in the heterogeneous network, KM^*^(*i, j*) represents the weight of the directed edge from the microbe node *m*_*i*_ to the microbe node *m*_*j*_, while KM^*^(*j, i*) denotes the weight of the directed edge from the microbe node *m*_*j*_ to the microbe node *m*_*i*_.

(8) $$KD^{*}(i,j)=\frac{KD(i,j)}{\sum_{i=1}^{N_{d}}KD(i,j)}\times NZ(d(i))$$

Where *NZ*[*d*(*i*)] denotes the number of elements with non-zero values in the *ith* row of the matrix *KD*. And based on the above formula (8), it is noteworthy that the symmetric matrix *KD* will as well be changed to an asymmetric matrix KD^*^ after the normalization. Moreover, in the heterogeneous network, KD^*^(*i, j*) represents the weight of the directed edge from the disease node *d*_*i*_ to the disease node *d*_*j*_, while KD^*^(*j, i*) denotes the weight of the directed edge from the disease node *d*_*j*_ to the disease node *d*_*i*_.

Therefore, according to the above descriptions, it is obvious that we can obtain a bidirectional heterogeneous network based on the above formula (7) and formula (8).

### 3.4. Bidirectional Recommendation of Potential Associations

**Figure 2:**
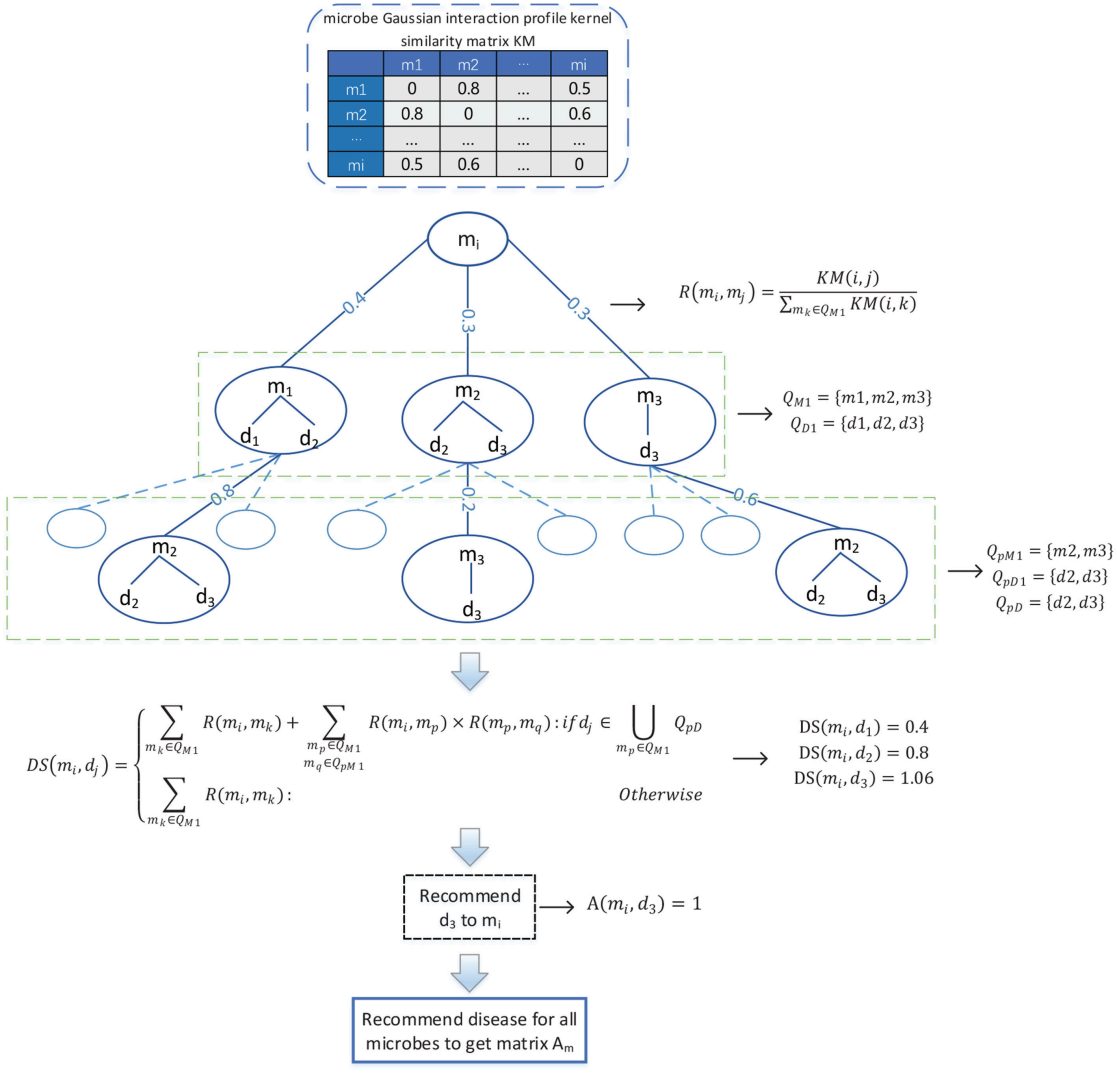
Flowchart of the method utilized to recommend diseases to microbes.

Considering that there are only 450 known associations in the adjacency matrix *A*, which is very sparse, therefore, in order to solve the problem of the adjacency matrix *A* caused by the scarcity of known associations, as illustrated in the following Figure 2, we designed a novel bidirectional recommendation model in this section based on the bidirectional heterogeneous network constructed above. And in this bidirectional recommendation model, we first designed a recommendation algorithm to recommend diseases for microbes based on the Gaussian interaction profile kernel similarities between microbes as follows:

(1) Firstly, for any given microbe node *m*_*i*_ in the bidirectional heterogeneous network, let *Q*_*M*1_ denote the set consisting of the first *K* microbes that are other than *m*_*i*_ in the bidirectional heterogeneous network and most similar to *m*_*i*_ at the same time, and considering about the time complexity, in this paper, *K* will be set to 3. And then, let *Q*_*D*1_ represent the set of diseases having known associations with at least one of the microbe nodes in *Q*_*M*1_, thereafter for any microbe node *m*_*j*_ in *Q*_*M*1_, we can obtain the recommendation score of *m*_*j*_ to *m*_*i*_ according to the following formula (9):

(9) $$\begin{array}{r} R\left(m_{i},m_{j}\right)=\frac{KM\left(i,j\right)}{\underset{m_{k}\in Q_{M1}}{\sum }KM\left(i,k\right)} \end{array}$$

Moreover, for any given disease node *d*_*j*_ in *Q*_*D*1_, we can further obtain the recommendation score of *d*_*j*_ to *m*_*i*_ according to the following formula (10):

(10) $$\begin{array}{r} DS\left(m_{i},d_{j}\right)=\underset{m_{k}\in Q_{M1}}{\sum }R\left(m_{i},m_{k}\right) \end{array}$$

Hence, in a similar way, for any given microbe node *m*_*p*_ in *Q*_*M*1_, we can obtain a set *Q*_*pM*1_ consisting of the first *K* microbes that are other than *m*_*p*_ in the bidirectional heterogeneous network and most similar to mp at the same time, and then, based on the set *Q*_*pM*1_, we can further obtain a set *Q*_*pD*1_ consisting of diseases that have known associations with at least one of the microbe nodes in *Q*_*pM*1_. In addition, let *Q*_*pD*_ = *Q*_*D*1_∩*Q*_*pD*1_, it is obvious that for any node *d*_*k*_ in ∪_*m*_*p*_∈*Q*_*M*1__*Q*_*pD*_, it shall be assigned higher recommendation score than those nodes that are in *Q*_*D*1_ and not in ∪_*m*_*p*_∈*Q*_*M*1__*Q*_*pD*_. Hence, for any given disease node *d*_*j*_ in *Q*_*D*1_, based on the above formula (10), we can obtain a modified recommendation score of *d*_*j*_ to *m*_*i*_ as follows:

(11) $$DS(m_{i},d_{j})=\{\begin{array}{c} \sum_{m_{k}\in Q_{M1}}R(m_{i},m_{k})+\sum_{\begin{array}{c} m_{p}\in Q_{M1}\\ m_{q}\in Q_{pM1} \end{array}}R(m_{i},m_{p})\times R(m_{p},m_{q}):ifd_{j}\in \underset{m_{p}\in Q_{M1}}{\cup }Q_{pD}\\ \sum_{m_{k}\in Q_{M1}}R(m_{i},m_{k}):otherwise \end{array}$$

Obviously, according to the above formula (11), for all these disease nodes in *Q*_*D*1_, we can obtain their corresponding recommendation scores, after sorting these disease nodes according to their recommendation scores in descending order, we will finally recommend the disease node ranking first to the microbe node *m*_*i*_. And additionally, for the microbe node *m*_*i*_, supposing that the disease node that we recommended to it is *d*_*j*_, then we will further set the value of *A*(*i, j*) in the adjacency matrix *A* to 1. Consequently, through updating the adjacency matrix *A* as stated above, it is obvious that we can obtain a new adjacency matrix *A*_*m*_.

(2) Secondly, in a similar way, for any given disease node *d*_*i*_ in the bidirectional heterogeneous network, let *Q*_*D*2_ denote the set consisting of the first *K* (=3) diseases that are other than *d*_*i*_ in the bidirectional heterogeneous network and most similar to *d*_*i*_ at the same time, and then, let *Q*_*M*2_ represent the set of microbes having known associations with at least one of the disease nodes in *Q*_*D*2_, thereafter, for any given disease node *d*_*p*_ in *Q*_*D*2_, we can obtain a set *Q*_*pD*2_ consisting of the first *K* diseases that are other than *d*_*p*_ in the bidirectional heterogeneous network and most similar to *d*_*p*_ at the same time. Moreover, based on the set *Q*_*pD*2_, we can further obtain a set *Q*_*pM*2_ consisting of microbes that have known associations with at least one of the disease nodes in *Q*_*pD*2_. Finally, let *QpM* = *Q*_*M*2_∩*Q*_*pM*2_, then for any given microbe node *m*_*j*_ in *Q*_*M*2_, we can obtain a recommendation score of *m*_*j*_ to *d*_*i*_ as follows:

(12) $$DS(d_{i},m_{j})=\{\begin{array}{c} \sum_{d_{k}\in Q_{D2}}R(d_{i},d_{k})+\sum_{\begin{array}{c} d_{p}\in Q_{D1}\\ d_{q}\in Q_{pD2} \end{array}}R(d_{i},d_{p})\times R(d_{p},d_{q}):ifm_{j}\in \underset{d_{p}\in Q_{D2}}{\cup }Q_{pM}\\ \sum_{d_{k}\in Q_{D2}}R(d_{i},d_{k}):otherwise \end{array}$$

Here,

(13) $$R(d_{i},d_{j})=\frac{KD(i,j)}{\sum_{d_{k}\in Q_{D2}}KD(i,k)}$$

Obviously, according to the above formula (12), for all these microbe nodes in *Q*_*M*2_, we can obtain their corresponding recommendation scores, after sorting these microbe nodes according to their recommendation scores in descending order, we will finally recommend the microbe node ranking first to the disease node *d*_*i*_. And additionally, for the disease node *d*_*i*_, supposing that the microbe node that we recommended to it is *m*_*j*_, then we will further set the value of *A*(*j, i*) in the adjacency matrix *A* to 1. Consequently, through updating the adjacency matrix *A* as stated above, it is obvious that we can obtain a new adjacency matrix *A*_*d*_.

### 3.5. Prediction Model of BWNMHMDA

KATZ is a network-based method that can solve link prediction problems. In recent years, KATZ has been implemented successfully in many different prediction applications such as prediction of social networks (Katz, 1953), prediction of associations between gene (Yang et al., 2014) and prediction of associations between lncRNAs (Chen, 2015), etc. In 2017, Chen et al. further applied KATZ in the field of microbe-disease association prediction for the first time (Chen et al., 2017). Considering that KATZ can be utilized to calculate the similarities between nodes in heterogeneous networks, and according to the above description in section 3.3, we have built a bidirectional heterogeneous microbe-disease association network, hence, in this section, we will design a model called BWNMHMDA based on KATZ to predict potential microbe-disease associations. For constructing the prediction model, we will convert the bidirectional heterogeneous microbe-disease association network to a (39+292)*(39+292) dimensional matrix *S* as follows:

(14) $$\begin{array}{r} S=\left[\begin{array}{cc} KD^{*} & A_{d}\\ A_{m}^{T} & KM^{*} \end{array}\right] \end{array}$$

Hence, based on above formula (14), for any given disease node *d*_*i*_ and microbe node *m*_*j*_ in the bidirectional heterogeneous microbe-disease association network, we can predict the potential similarity between them as follows:

(15) $$\begin{array}{r} Sim\left(d_{i},m_{j}\right)=A_{n}^{*}\left(i,j\right) \end{array}$$

Here, *n* is a parameter representing the number of steps between disease nodes and microbe nodes in the bidirectional heterogeneous microbe-disease association network. For *n* = 1, 2, 3, …, there are:

(16) $$\begin{array}{rcl} A_{n}^{*} & = & \frac{S_{n2}+S_{n3}^{T}}{2} \end{array}$$

(17) $$\begin{array}{rcl} S_{n} & = & S_{n-1}\times W\times S_{n-1}=\left[\begin{array}{cc} S_{n1} & S_{n2}\\ S_{n3} & S_{n4} \end{array}\right] \end{array}$$

(18) $$\begin{array}{rcl} S_{2} & = & S\times W\times S \end{array}$$

Specifically, in formula (16), the matrix *S*_*n*2_(*i, j*) represents the total score of all paths with length of *n* from the disease *d*_*i*_ to microbe *m*_*j*_, and correspondingly, the matrix *S*_*n*3_(*j, i*) represents the total score of all paths with length of *n* from the microbe *m*_*j*_ to disease *d*_*i*_. It is worth noting that since the weights of the edges in the heterogeneous network are bidirectional, we integrate *S*_*n*2_ and *S*_*n*3_ as formula (16). The two matrices are assigned the same weight as the final predictive score matrix $A_{n}^{*}$.

## 4. Result

### 4.1. Effects of the Parameter *n* to BWNMHMDA

The framework of Leave-one-out cross validation (LOOCV) and 5-fold cross validation (5-Fold CV) are two kinds of common methods to evaluate model performance. While implementing LOOCV on our prediction model BWNMHMDA, each known microbe-disease association will be used as a test sample and further predicted by training the other known microbe-disease associations. Moreover, all microbe-disease pairs without known relevant evidence will be considered as candidate samples. The predicted score which obtained a higher rank than the given threshold will be considered as a successful prediction. Obviously, while setting different thresholds, the true positive rate (TPRs, sensitivity) and false positive rate (FPRs, 1-specificity) can be obtained. Here, sensitivity refers to the percentage between the number of test samples with ranks higher than the given threshold and the number of positive samples (known microbe-disease associations). Meanwhile, 1-specificity denotes the percentage of negative microbe-disease associations which obtained ranks lower than the threshold. Finally, the receiver operating characteristic (ROC) curve can be further drawn. The area under the ROC curve(AUC) can be calculated to evaluate its predictive performance, where the AUC value of 1 indicates perfect prediction perfection and the AUC value of 0.5 implies pure random prediction performance (Chen et al., 2017).

**Figure 3:**
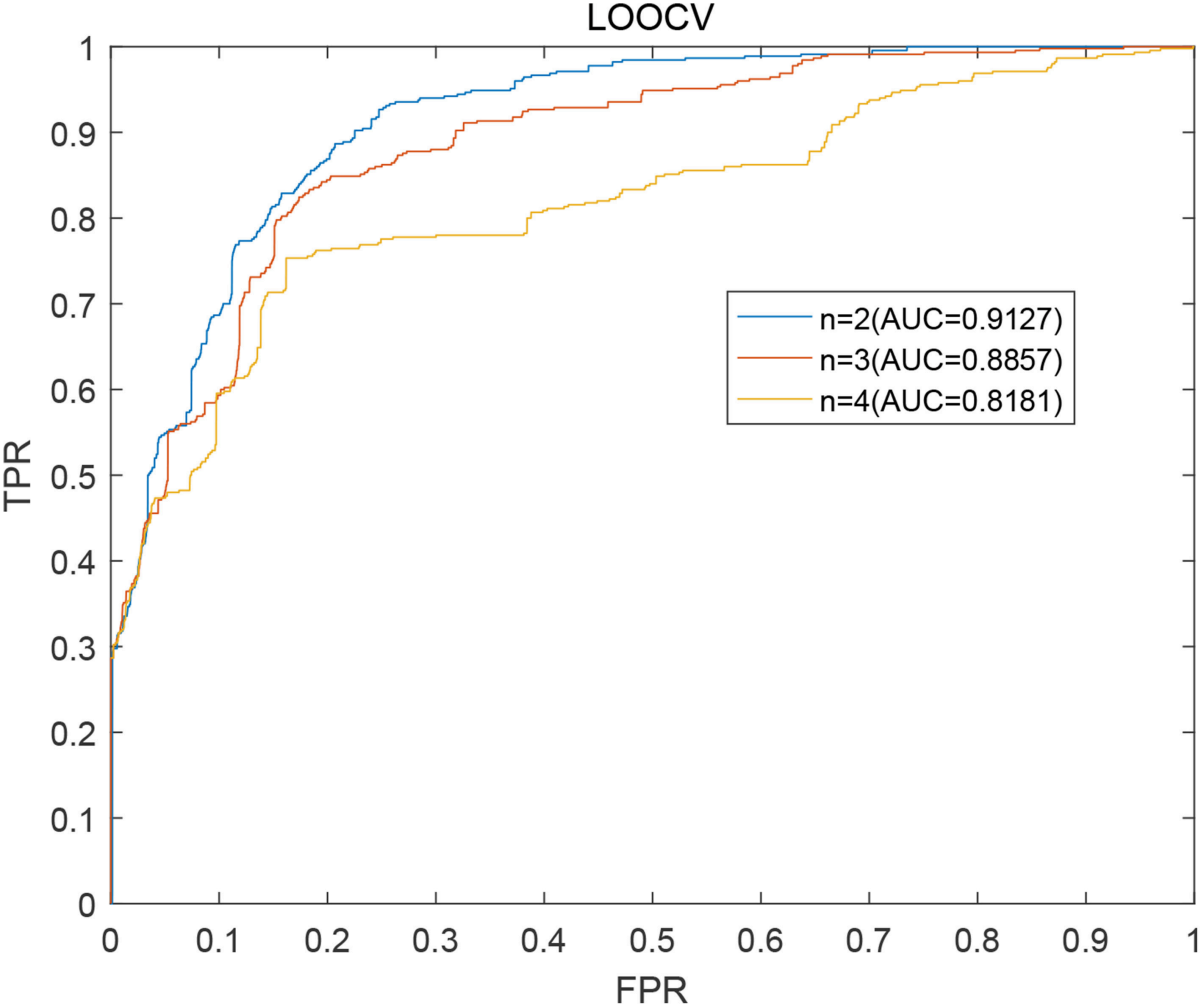
AUCs achieved by BWNMHMDA in LOOCV while *n* = 2, 3, 4 separately.

As described above, in our prediction model BWNMHMDA, the variable *n* in the formulas (15) is a critical parameter. Hence, we will first estimate its effect to the prediction performance of BWNMHMDA in this section. And as illustrated in Figure 3. BWNMHMDA achieved the best prediction performance while *n* = 2, and as the value of *n* sequentially increased from 2 to 4, the AUCs achieved by BWNMHMDA decreased continuously, and through analysis, we found that the reason may be that the number of known microbe-disease associations is minimal in the HMDAD database, which leads that long paths in the bidirectional heterogeneous microbe-disease association network will be meaningless to the prediction performance of BWNMHMDA.

In order to further evaluate the effects of the parameter *n* to our prediction model, we further implemented 5-fold cross validation on BWNMHMDA, and during simulation, all known microbe-disease associations were randomly divided into five segments with almost the same size, among which, four segments were utilized for model learning, and the remaining segment were used as test samples for model evaluation. Similar to LOOCV, all microbe-disease pairs without relevant evidence would be considered as potential candidates. In order to reduce the experimental bias, we repeated our simulation based on the 5-fold cross validation 100 times, and during each time of simulation, the samples were divided randomly. Finally, as illustrated in the following Table 1, it is easy to see that BWNMHMDA could as well achieve the best prediction performance while *n*=2, and moreover, as the value of *n* sequentially increased from 2 to 4, the AUCs achieved by BWNMHMDA also decreased continuously. Hence, we will set *n* to 2 in the subsequent experiments.

**Table 1:** AUCs achieved by BWNMHMDA in the framework of 5-Fold CV while *n* = 2, 3, 4 separately.

***n* = 2**	***n* = 3**	***n* = 4**
0.8967 ± 0.0027	0.8804 ± 0.0026	0.8109 ± 0.0052

### 4.2. Comparison With Other State-of-the-Art Methods

**Figure 4:**
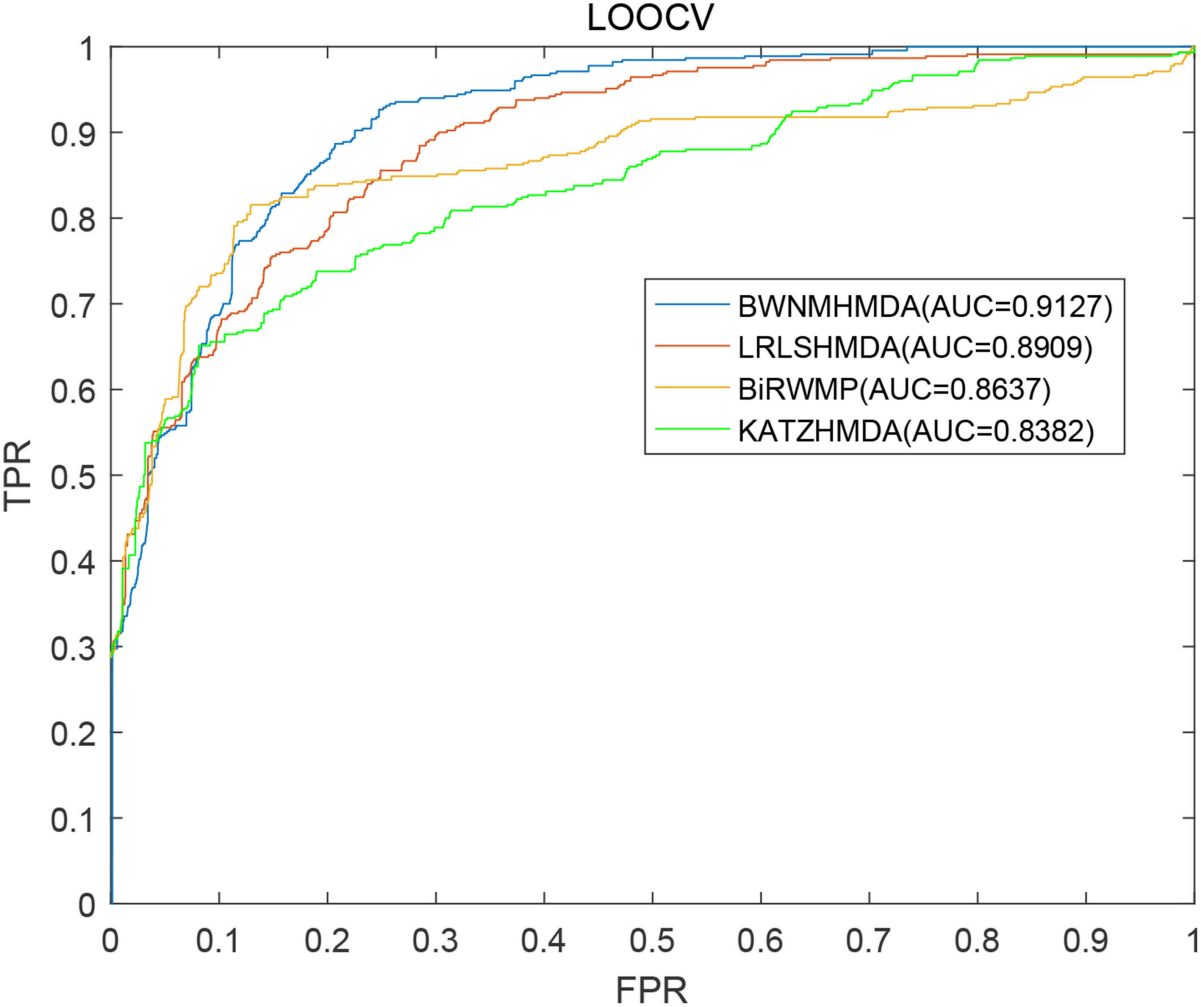
AUCs achieved by KATZHMDA, BiRWMP, LRLSHMDA, and BWNMHMDA in LOOCV.

In order to verify the prediction performance of BWNMHMDA, in this section, we compared it with KATZHMDA (Chen et al., 2017), BiRWMP (Shen et al., 2018), and LRLSHMDA (Wang et al., 2017) based on the dataset of known microbe-disease associations downloaded from the HMDAD database. And as illustrated in the following Figure 4 and Table 2, it is easy to see that in LOOCV, BWNMHMDA can achieve a reliable AUC of 0.9127 that is much better than the AUC achieved by KATZHMDA (0.8382), BiRWMP (0.8637), and LRLSHMDA (0.8909), and in the framework of 5-fold cross validation, BWNMHMDA can achieve a reliable AUC of 0.8967 ± 0.0027 that is much better than the AUC achieved by KATZHMDA (0.8301 ± 0.0033), BiRWMP (0.8522 ± 0.0054), and LRLSHMDA (0.8794 ± 0.0029) as well.

**Table 2:** AUCs achieved by BWNMHMDA, KATZHMDA, BiRWMP, and LRLSHMDA in LOOCV and 5-Fold CV separately.

**Method**	**LOOCV**	**5-Fold CV**
BWNMHMDA	0.9127	0.8967 ± 0.0027
LRLSHMDA	0.8909	0.8794 ± 0.0029
BiRWMP	0.8637	0.8522 ± 0.0054
KATZHMDA	0.8382	0.8301 ± 0.0033

We further compare BWNMHMDA with NGRHMDA (Huang Y.A. et al., 2017), ABHMDA (Peng et al., 2018), and BMCMDA (Shi et al., 2018) in LOOCV based on the same dataset. As shown in Table 3, our method achieves the best performance.

**Table 3:** AUCs achieved by BWNMHMDA, NGRHMDA, ABHMDA, and BMCMDA in LOOCV separately.

**Method**	**BWNMHMDA**	**NGRHMDA**	**ABHMDA**	**BMCMDA**
AUC	0.9127	0.8938	0.8869	0.906

## 5. Case Studies

In order to further measure the prediction performance of BWNMHMDA, in this section, we selected three kinds of important human diseases such as asthma, colorectal carcinoma, and COPD (Chronic Obstructive Pulmonary Disease) to explore the associations between the human microbes and the human respiratory and digestive system diseases. Among them, asthma is a heterogeneous disease process accompanied by recurrent episodes of wheezing, chest tightness, difficulty breathing, and indirect cough (Busse, 2007). In recent years, the prevalence of asthma is rising rapidly. It is reported that about 8% of people have been affected by asthma by 2010, especially in the children's population (Guilbert et al., 2014). Hence, considering that asthma has been demonstrated to be closely associated with microbes as well (Çalşkan et al., 2013; Gilstrap and Kraft, 2013), for example, *Hemophilia, Moraxella*, and *Neisseria* spp. in the lungs of asthma patients are proved to be closely related to the increased risk of asthma in the neonatal oropharynx. *Staphylococcus* was found in the respiratory tract of children with asthma (Sullivan et al., 2016), in this section, we selected asthma as one of our case studies to evaluate the performance of BWNMHMDA. And as illustrated in the following Table 4, all of these top 10 microorganisms predicted by BWNMHMDA have been verified to be associated with the onset of asthma. For example, *Tropheryma whipplei* (Ranking first in the list of top 10 predicted microbes) has been confirmed to be abundant in airway of patients with eosinophilic asthma (Simpson et al., 2015). *Clostridium difficile* (Ranking second in the list of top 10 predicted microbes) has been confirmed to be associated with asthma after 6–7 years of colonization (van Nimwegen et al., 2011). *Firmicutes* (Ranking third in the list of top 10 predicted microbes) has been confirmed to be increased in severe asthmatics (Zhang et al., 2016). Furthermore, the increased sensitivity to *Staphylococcus aureus* (Ranking fifth in the list of top 10 predicted microbes) has been proved to be a marker of eosinophilic inflammation and severe asthma in asthmatic patients as well (Nagasaki et al., 2017). We published evidence for the top 10 potential asthma-related microbes predicted by BWNMHMDA in the Table 4.

**Table 4:** Top 10 potential asthma-related microbes predicted by BWNMHMDA and all of these 10 microbes have been confirmed by evidences.

**Rank**	**Microbe**	**Evidence**
1	*Tropheryma whipplei*	PMID: 26647445
2	*Clostridium difficile*	PMID: 21872915
3	*Firmicutes*	PMID: 27078029
4	*Lachnospiraceae*	PMID: 26512904
5	*Staphylococcus aureus*	PMID: 17950502
6	*Clostridia*	PMID: 22047069
7	*Bacteroides*	PMID: 18822123
8	*Fusobacterium*	PMID: 24024497
9	*Clostridium coccoides*	PMID: 21477358
10	*Actinobacteria*	PMID: 23265859

In recent years, colorectal carcinoma (CRC) is becoming a major cause of cancer mortality in both China and the United States. In 2016, an estimated 134,000 people had been diagnosed with CRC, and approximately 49,000 had died of CRC (Bibbins-Domingo et al., 2008). By gender, CRC is the second most common cancer in women (about 9.2%) and the third in men (about 10%) (Astin et al., 2011). Since it has been proved that CRC is related to gut microbiota such as the *Fusobacterium*, the *Bacteroides fragilis* and the enteropathogenic *Escherichia coli*, and the dysbiosis of these gut microbiotas will induce colon cancer through a chronic inflammatory mechanism (Mármol et al., 2017). Hence in this section, we selected CRC as one of our case studies to evaluate the performance of BWNMHMDA. And as illustrated in the following Table 5, there are 9 out of these top 10 microorganisms predicted by BWNMHMDA have been verified to be associated with the onset of colorectal carcinoma. For instance, related studies have shown that the abundance of *Firmicutes* (Ranking 6th in the list of top 10 predicted microbes) in the lumen of CRC rats will increase, while the abundance of *Bacteroidetes* (Ranking 4th in the list of top 10 predicted microbes) will reduce. And moreover, the abundance of *Proteobacteria* (Ranking second in the list of top 10 predicted microbes) has been confirmed to be higher in CRC rats than in healthy rats. Meanwhile, *Bacteroides* (Ranking 9th in the list of top 10 predicted microbes) has been proved to of a relatively high abundance in CRC rats at the genus level. *Prevotella* (Ranking third in the list of top 10 predicted microbes) has been found to be significantly more abundant in healthy rats than CRC rats (Zhu et al., 2014). Additionally, compared with the healthy control group, Fukugaiti MH et al. detected more *C. difficile* (Ranking 5th in the list of top 10 predicted microbes) in the cancer group, which suggests that these bacteria may play an important role in the colorectal carcinoma (Fukugaiti et al., 2015). We published evidence for the top 10 potential CRC-related microbes predicted by BWNMHMDA in the Table 5.

**Table 5:** Top 10 potential CRC-related microbes predicted by BWNMHMDA and 9 out of these 10 microbes have been confirmed by evidences.

**Rank**	**Microbe**	**Evidence**
1	*Tropheryma whipplei*	Unconfirmed
2	*Proteobacteria*	PMID:24603888
3	*Prevotella*	PMID:29432368
4	*Bacteroidetes*	PMID:26992426
5	*Clostridium difficile*	PMID:19807912
6	*Firmicutes*	PMID:29985435
7	*Helicobacter pylori*	PMID:11774957
8	*Clostridia*	PMID:26691472
9	*Bacteroides*	PMID:30090033
10	*Staphylococcus aureus*	PMID:7074582

Finally, COPD is an obstructive pulmonary disease that worsens over time, and the main symptoms of COPD are shortness of breath and coughing. And as of 2015, patients with chronic obstructive pulmonary disease accounted for approximately 174.5 million (about 2.4%) of the global population (Vos et al., 2016). For the past few years, due to high smoking rates and an aging population in developing countries, the death toll of COPD is rising fast (Mathers and Loncar, 2006). Although treatments can slow the progression of COPD, there is no cure yet. Considering that many evidences have demonstrated that there exist associations between microbiomes and COPD, for instance, Galiana et al. found that the microbiota diversity of patients with severe COPD was lower than that of mild/moderate diseases, and actinomyces accounted for a high proportion of patients with severe COPD (Galiana et al., 2013), hence in this section, we selected COPD as one of our case studies to evaluate the performance of BWNMHMDA. And as illustrated in the following Table 6, there are 8 out of these top 10 microorganisms predicted by BWNMHMDA have been verified to be associated with the onset of COPD. For instance, COPD has been confirmed to be a kind of essential comorbidity in human immunodeficiency virus (HIV) patients, and more *T. whipplei* (Ranking first in the list of top 10 predicted microbes) has found in lower airway of human immunodeficiency virus-infected subjects (Segal et al., 2014; Sze et al., 2016). And also, it has been demonstrated that *Proteobacteria* (Ranking second in the list of top 10 predicted microbes) and *Firmicutes* (Ranking 3rd in the list of top 10 predicted microbes) will increase significantly with the development of COPD (Pragman et al., 2012). We published evidence for the top 10 potential COPD-related microbes predicted by BWNMHMDA in the Table 6.

**Table 6:** Top 10 potential COPD-related microbes predicted by BWNMHMDA and 8 out of these 10 microbes have been confirmed by evidences.

**Rank**	**Microbe**	**Evidence**
1	*Tropheryma whipplei*	PMID:24460444
2	*Proteobacteria*	PMID:23071781
3	*Bacteroidetes*	PMID:29709671
4	*Prevotella*	PMID:30053882
5	*Clostridium difficile*	PMID:15655746
6	*Firmicutes*	PMID:24591822
7	*Helicobacter pylori*	PMID:15733502
8	*Lachnospiraceae*	Unconfirmed
9	*Staphylococcus aureus*	Unconfirmed
10	*Clostridia*	PMID:26852737

Furthermore, in order to reconfirm the prediction performance of BWNMHMDA, we compared it with KATZHMDA in the case studies of these three kinds of same diseases, and as shown in the following Table 7, it is obvious that there are 10, 9, and 8 out of these top 10 microbes predicted by BWNMHMDA having been verified to be associated with the onset of asthma, colorectal carcinoma and COPD respectively, while there are only 4, 5, and 5 out of these top 10 microbes predicted by KATZHMDA having been verified to be associated with the onset of asthma, colorectal carcinoma, and COPD separately, which demonstrated that our prediction model BWNMHMDA could achieve better predictive hit rate in case above studies than the prediction model of KATZHMDA. And in addition, we published all these rankings of microbe-disease associations and top 10 disease-related microbes predicted by BWNMHMDA in Supplementary Tables 1, 2, respectively, and hope that these data may provide some help to the future works of relevant researchers.

**Table 7:** The number of of microbes having been confirmed by evidences in the top 10 potential disease-related microbes predicted by BWNMHMDA and KATZHMDA respectively in case studies of the three kinds of diseases such as Asthma, CRC, and COPD.

**Model**	**Asthma**	**colorectal carcinoma**	**COPD**
BWNMHMDA	10	9	8
KATZHMDA	4	5	5

## 6. Discussion and Conclusion

Human microbiome is normal flora for humans, which has been proved to be of symbiotic relationship with humans and harmless to humans. If the microbes that breed in the human body become “unhealthy,” it will definitely affect the host's physical condition. People are continuing to explore the pathologic relationship between microorganisms and the human body through high-throughput sequencing technologies and analysis systems. However, it is a pity that their pathogenesis cannot be fully understood as yet. Considering that relying only on conventional experimental methods is time-consuming and laborious, in this article, we proposed a novel prediction model called BWNMHMDA to accelerate the process of inferring potential microbe-disease associations, in which, the core idea is to construct a weighted bidirectional microbe-disease association network and then convert it into a matrix for correlation probability calculation. While constructing the prediction model BWNMHMDA, we first downloaded known microbe-disease associations from the HDMDA database, and then, based on these downloaded associations, we constructed a heterogeneous network through adopting the Gaussian interaction profile kernel similarity to calculate the weights of nodes in the heterogeneous network. Moreover, based on the heterogeneous network, we further constructed a weighted bidirectional network by standardizing the weights of edges in the heterogeneous network and introducing a novel bidirectional recommendation method. Finally, we transformed the weighted bidirectional network into an integration matrix that can be utilized for prediction of potential microbe-disease associations. And simulation results show that BWNMHMDA can achieve reliable AUCs of 0.9127 and 0.8967 ± 0.0027 in the frameworks of LOOCV and 5-Fold CV respectively. And moreover, in the case studies of asthma, colorectal cancer, and COPD, there are 10, 9, and 8 out of the top 10 potential associated microbes predicted by BWNMHMDA having been verified by published literature evidence, which demonstrated that BWNMHMDA could provide valuable potential microbe-disease associations for future biological experiments. Certainly, there are some deficiencies in BWNMHMDA. For instance, there is a lack of negative samples in BWNMHMDA, and it may be possible to improve the predictive reliability of BWNMHMDA by identifying unrelated microbe-disease pairs. And moreover, in BWNMHMDA, we adopt the Gaussian interaction profile kernel similarity to calculate the similarities between microbes, which may bias the similarity between some individual microbes. Hence, in subsequent work, we will introduce some effective methods such as Symptom-Based Disease Similarity (Zhou et al., 2014) to further improve the accuracy and efficiency of BWNMHMDA.

## Author Contributions

HL and LW conceptualized the study. HL and YW created the methodology, conducted the validation, and the data curation. HL, YW, HZ, and LW conducted the formal analysis. JJ, XF, HZ, and BZ oversaw the investigations. HL provided resources and prepared and wrote the original draft. LW wrote, reviewed and edited the manuscript, supervised the project, oversaw project administration, and acquired funding.

### Conflict of Interest Statement

The authors declare that the research was conducted in the absence of any commercial or financial relationships that could be construed as a potential conflict of interest.

## Supplementary Material

Supplementary Table 1 Ranks of microbe-disease associations predicted by BWNMHMDA.

Supplementary Table 2 Top 10 related microbes of all diseases.
